# Is sentinel node mapping useful for esophageal cancer surgery?

**DOI:** 10.1002/ags3.12231

**Published:** 2019-01-15

**Authors:** Soji Ozawa

**Affiliations:** ^1^ Department of Gastroenterological Surgery Tokai University School of Medicine Kanagawa Japan

Sentinel node (SN) mapping has been used to obtain important information for selective lymphadenectomy in patients with early‐stage melanoma and breast cancer.^1^, ^2^ SN are defined as the first draining lymph nodes for the primary tumor and are, therefore, the first possible sites of micrometastasis along the lymphatic drainage route from the primary tumor site. Histopathological examination of SN is considered to be useful for predicting the degree of lymph node metastasis. When SN are identified and confirmed to be free of cancer cells, standard lymphadenectomy can be avoided.

Regarding patients with upper gastrointestinal cancer, many studies have reported that SN mapping yields a good detection rate and diagnostic accuracy for determining lymph node status in patients with early‐stage esophageal and gastric cancer. Avoidance of unnecessary lymphadenectomy and reducing the extent of organ resection would inevitably result in a better quality of life postoperatively.

Esophageal cancer is one of the most malignant of all gastroenterological cancers, and lymph node metastasis is a significant prognostic factor. Because of anatomical reasons, it is associated with a complicated pattern of lymph node metastasis, from the cervical to the abdominal region. Lymph node metastasis occurs in approximately 40% of cases of early‐stage (T1b) esophageal cancer. Therefore, esophagectomy with two‐ or three‐field lymphadenectomy is considered a standard surgical procedure, even for cN0 esophageal cancer.^3^ Pneumonia and recurrent laryngeal nerve palsy were observed as postoperative complications in 14% and 10% of cases, respectively.^4^ To reduce the incidence of these complications, less invasive esophagectomy procedures have been sought. It would be of interest to most surgeons operating on esophageal cancer patients to know whether less invasive surgery for early‐stage esophageal cancer is possible using SN mapping.

In this issue of the *Annals of Gastroenterological Surgery*, Takeuchi et al present a nice review of SN navigation surgery in esophageal cancer. As for SN mapping and biopsy procedures in esophageal cancer, a radioisotope‐guided method is recommended for the detection of SN in cases of thoracic esophageal cancer. ^99m^Sn colloid solution, as the radioisotope tracer, is injected into the submucosal layer in the four quadrants surrounding the primary tumor. Preoperative lymphoscintigraphy and intraoperative detection of the SN using a handheld gamma probe are usually carried out. After intraoperative SN mapping and biopsy, all the SN in the resected specimens are confirmed using the handheld gamma probe and examined histopathologically. A dual‐tracer method using a radioactive tracer and blue dye is useful for SN detection in patients with abdominal esophageal cancer or adenocarcinoma of the esophagogastric junction. Dye‐only‐guided SN mapping is not recommended for thoracic esophageal cancer because of a poor detection rate. The radioisotope‐guided method is superior in terms of SN detection rate and accuracy at predicting lymph node metastasis as compared to the conventional dye‐guided method in patients with esophageal cancer.

Regarding the influence of tumor depth, although cT1 esophageal cancers are suitable for SN mapping, T3/T4 tumors are not suitable, because the original lymphatic drainage routes may be altered in the latter. It remains controversial as to whether SN mapping is possible for patients who have received neoadjuvant chemoradiotherapy, considering the high false‐negative rate reported. Distribution of SN in cases of esophageal cancer is widespread, from the cervical to the abdominal areas. In cases of upper thoracic esophageal cancer, SN are identified in the lymph nodes along the bilateral recurrent laryngeal nerve chain and lymph nodes in the cervical area. In cases of middle thoracic esophageal cancer, SN are widely distributed from the cervical to the abdominal region. In cases of lower thoracic esophageal cancer, SN are mainly located in the lower and middle mediastinum and in the abdominal area. A systematic review and meta‐analysis showed that SN detection rate and accuracy of prediction of lymph node metastasis based on SN status were 93% and 88%, respectively, and not markedly different between squamous cell carcinoma and adenocarcinoma. It is considered that SN mapping is technically feasible in esophageal cancer.

Some possible scenarios for omission of standard lymphadenectomy using SN mapping in patients with esophageal cancer are as follows. In patients with cT1N0M0 middle or lower thoracic esophageal cancer, when SN are detected in the mediastinum or abdominal area only and all the SN are histopathologically negative for cancer metastasis, cervical lymphadenectomy can be omitted. In patients with cT1N0M0 adenocarcinoma of the distal esophagus, when all the SN are identified in the abdominal area only and all the SN are histopathologically negative for cancer metastasis, limited resection of the distal esophagus can be carried out by a laparoscopic transhiatal approach without extensive mediastinal lymphadenectomy. Recently, transmediastinal esophagectomy has been carried out and been shown to be associated with a lower incidence of postoperative pneumonia.^5^ In patients with cT1N0M0 thoracic esophageal cancer, when all the SN are histopathologically negative for cancer metastasis, additional lymphadenectomy might be omitted while carrying out transmediastinal esophagectomy.

Sentinel node mapping may be useful for developing new surgical methods, in terms of carrying out appropriate lymphadenectomy, and in patients with cT1N0M0 esophageal cancer.

## DISCLOSURE

Conflicts of Interest: Author declares no conflicts of interest for this article.
